# Development of Autophagy Signature-Based Prognostic Nomogram for Refined Glioma Survival Prognostication

**DOI:** 10.1155/2020/1872962

**Published:** 2020-09-04

**Authors:** Yuxiang Fan, Xinyu Peng, Baoqin Li, Gang Zhao

**Affiliations:** ^1^Department of Neurosurgery, The First Hospital of Jilin University, Changchun 130021, China; ^2^Department of Hepatobiliary Pancreatic Surgery, The First Hospital of Jilin University, Changchun 130021, China; ^3^Department of Spine Surgery, The First Hospital of Jilin University, Changchun 130021, China

## Abstract

The current glioma classification could be optimized to cover such a separate and individualized prognosis ranging from a few months to over ten years. Considering its highly conserved role and potential in therapies, autophagy might be a promising element to be incorporated as a refinement for improved survival prognostication. The expression and RNA-seq data of 881 glioma patients from the Gene Expression Omnibus and The Cancer Genome Atlas were included, mapped with autophagy-related genes. Weighted gene coexpression network analysis and Cox regression analysis were used for the autophagy signature establishment, which composed of *MUL1*, *NPC1*, and *TRIM13*. Validations were represented by Kaplan-Meier plots and receiver operating curves (ROC). Cluster analysis suggested the *IDH1* mutant involved in the favorable prognosis of the signature clusters. The signature was also immune-related shown by the Gene Ontology analysis and the Gene Set Enrichment Analysis. The high signature risk group held a higher ESTIMATE score (*p* = 2.6*e* − 11) and stromal score (*p* = 1.8*e* − 10). CD276 significantly correlated with the signature (*r* = 0.51, *p* < 0.05). The final nomogram integrated with the autophagy signature, *IDH1* mutation, and pathological grade was built with accuracy and discrimination (1-year survival AUC = 0.812, 5-year survival AUC = 0.822, and 10-year survival AUC = 0.834). Its prognostic value and clinical utility were well-defined by the superiority in the comparisons with the current World Health Organization glioma classification in ROC (*p* < 0.05) and decision curve analysis. The autophagy signature-based *IDH1* mutation and grade nomogram refined glioma classification for a more individualized and clinically applicable survival estimation and inspired potential autophagy-related therapies.

## 1. Introduction

Glioma, accounting for the majority of malignant primary brain tumors in adults, earns the prestige due to its “bipolar” prognosis. Typically, its median survival spans from nearly one-year survival of glioblastoma multiforme (WHO IV) to over ten years with a 90% chance if diagnosed with pilocytic astrocytoma (WHO I) [1]. Less competence of microscopic morphology grading in covering such a separate prognosis permits the molecular testing era to arrive. With progress in genomics, a detailed stratification of gliomas for precise prognosis prediction and therapy decision-making is refined by the integration of conventional histopathological observations with genotypic alterations [2].

Isocitrate dehydrogenase (IDH) mutations, codeletion of chromosomal arms of 1p and 19q, O6-methylguanine-DNA methyltransferase (MGMT) promoter gene methylation, and histone protein H3.3 or H3.1lys27Met mutations (*H3K27M*) represent prevalent subtypes in the population with glioma [2]. It is widely accepted that patients with *IDH1*mutaions would likely enjoy more extended overall survival (OS) than their wildtype counterparts almost in each subtype of glioma [3]. *IDH1* mutation earns its reputation also based on the hypothesis that it is the mutation that might drive the lower grade glioma (LGG) trending towards malignancy [4]. Mutations like *IDH1* mutation, *1p/19q* codeletion, or methylation of MGMT promotor might favor prolonged survival and improved response to therapies, but they probably risk malignancy and even relapse for the protracted time. Given that not every single glioma patient showed mutations, let alone some rare mutations, further endeavors to incorporate not only mutations but also molecular signatures are warranted for more predictive, individualized, and therapy-guiding glioma classification.

Autophagy evolutionarily maintains cellular energy homeostasis by self-digesting labeled proteins and organelles through lysosomes. Reports considering autophagy in therapy resistance and crippling antitumor immune response have piled up [5, 6]. The fact that glioblastoma exploits autophagy as an adaption to temozolomide renders autophagy an intriguing target [7]. Minor tweaks with drug perturbation would convert autophagy from cytoprotective to cytotoxic role [8]. Autophagy impairs antitumor response by hindering monocyte differentiation into proinflammatory M1 macrophages, as well as T cells requiring autophagy to function and differentiate [9]. Additionally, macrophages in the peritumor microenvironment promote antitumor immunity when LC3-associated phagocytosis component genes are deleted [10]. Stands as a nexus in glioma, autophagy hereby lays a foundation for this study.

In this study, we attempted to build an autophagy signature with high-throughput data for better glioma survival prediction and underlying target gene screening. Initially, we assembled ATGs and searched for coexpression gene modules using weighted gene coexpression network analysis (WGCNA). The coexpression gene module most-related to OS would be selected and regarded as candidates for the risk gene signature construction. After validations, we also managed to interpret the signature by linking the model to the mutation stratification and by performing functional enrichment analysis. The final integrative nomogram model was not fully completed until the validations were accomplished. It was concluded that the autophagy signature-based *IDH1* mutation and tumor grade (AIM-g) nomogram fitted almost all of the glioma patients for more precise and personalized survival prognostication.

## 2. Materials and Methods

### 2.1. Microarray and Transcriptome Data with Autophagy-Related Gene Sets

The gene expression microarray dataset GSE16011 of glioma patients and its clinical traits were gathered from the Gene Expression Omnibus (GEO) database. The dataset recruited 284 glioma patients with histological stages including 117 cases of lower-grade glioma (LGG), 156 cases of glioblastoma multiforme (GBM), and normal controls [11]. Patients with clear mutation status were kept.

Of the glioma cohorts in The Cancer Genome Atlas (TCGA) database, 505 LGG and 155 GBM RNA-seq counts data with detailed clinical information were obtained with the R package “TCGAbiolinks” [12]. The corresponding mutation data was acquired by the R package “TCGAmutations” [13].

Several autophagy-related genes (*n* = 505) were selected in the Molecular Signatures Database v7.1 (MSigDB) [14]. After deduplicating with the whole gene set comprised of 232 genes in the (HADb) [15], a gene set of 598 genes was completed.

### 2.2. Construction of WGCNA in Autophagy-Related Gene Set

Weighted gene coexpression network analysis was constructed based on ATGs using the WGCNA package [16]. It helped calculate and transform a weighted adjacency matrix into a topological overlap matrix by employing the power (*β*) value as a threshold. Genes would be clustered with the others showing similar expression patterns and formed modules. The dynamic tree-cutting algorithm determined the ultimate modules.

Module eigengene (ME) was the first principal component set by the principal component analysis of each module. Module membership (MM) was thereafter determined by correlation analysis among genes in the module and the MEs. With correlation analysis implemented, genes coexpressed in modules might show their links to the clinical traits in GSE16011, including age, gender, histology, *IDH1* mutation status, and overall survival (OS). Overall survival was a period measured either from the day of glioma diagnosis or treatment to the end of one's time.

### 2.3. Prognostic Autophagy Signature Development and Validation

The correlation-promising and statistically significant modules were selected and regarded as resources for further screening of risk candidates. Univariate and multivariate Cox regression were orderly performed to measure the prognostic value of candidate genes building autophagy risk signature for OS prediction. The regression coefficients for each gene in the signature were utilized to calculate the autophagy signature risk score as follows, Risk score = ∑_*i*=1_^*n*^ *β*_*i*_ × gene_*i*_, where *β* indicated the Cox regression coefficient for the gene expression.

The time-dependent receiver operating curve (ROC) along with survival analysis was carried out for the model evaluation [17]. The GEO developing cohort was split at a ratio of 3 : 7 by the R package “caret” as the internal validation of the signature, in which the training set occupied 30% and the testing set took the rest [18]. The external validation also mainly relied on survival analysis and time-dependent ROC in the TCGA cohort.

### 2.4. Cluster and Mutation Analysis

Hierarchical clustering analysis was applied in both the GEO and the TCGA cohort. The clusters were cut into four simply according to four main branches of the clustering tree in the heat map. The mutation profile of each cluster grouped by the expression of the autophagy signatures was managed by the R package “Maftools” in the TCGA cohort [19].

### 2.5. Functional Enrichment Analysis

To explain functional gene sets underlying high- and low-risk groups, the Gene Ontology (GO) analysis was performed with the differentially expressed genes (DEGs) in the GEO cohort. DEGs were identified if adj-*p* less than 0.01 and |logFC| over cutoff value by the R package “limma” [20]. The mean of |logFC| adding two times standard deviation was deemed as the cutoff value. Gene set enrichment analysis (GSEA) was also conducted in the GSEA software (version 4.0.3) and visualized with the cystoscope software (version 3.7.2) [21–23]. In the GSEA results, it rendered statistical significance in functional enrichment when ∣*NES* | >1, NOM *p* value < 0.05, and FDR *q* value < 0.25.

### 2.6. ESTIMATE Immune Status in the Autophagy Signature Risk Groups

Estimation of STromal and Immune cells in MAlignant Tumor tissues using Expression data (ESTIMATE) analysis was utilized to evaluate the tumor purity in the tumor tissues [24]. It represented the immune score, stromal score, ESTIMATE score, and tumor purity as results. The detailed immune checkpoint gene correlation results were visualized by the R package “circlize” [25].

### 2.7. Nomogram Development and Validation

Autophagy signature, *IDH1* mutation, and pathological grade were tested and adjusted with multivariate Cox regression analysis. The AIM-g nomogram was then established using Cox regression in the R package “rms” [26]. It went through time-ROC analysis in the GEO and the TCGA cohort as assessments and verifications. Calibration curves were also used. Lastly, the comparisons between the AIM-g model and the WHO model (*IDH1* mutation and grade), concerning ROC at 1-, 3-, 5-, and 10-year time points, were conducted. Decision curve analysis (DCA) was performed for the clinical utility comparisons of the AIM-g nomogram with the WHO model [27].

### 2.8. Survival and Statistical Analysis

All the statistical analyses and graphs in this study were achieved in R 3.6.2 (R Core Team, 2019) and RStudio (version 1.1.463). Cox regression analysis, proportional hazards (PH) test, and Kaplan-Meier survival analysis were performed with the R package “survival” [28]. Sankey diagram was depicted to illustrate data structure via the package “ggalluvial” [29]. Wilcoxon test and Kruskal-Wallis test were used for statistical comparisons. Spearman tests were selected to test correlation. *p* value less than 0.05 was deemed statistical significance.

## 3. Results

**Figure 1:**
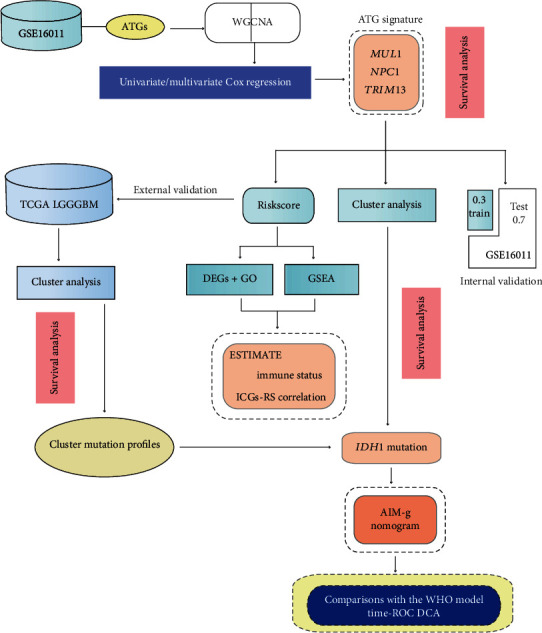
Framework of the study. ATG: autophagy-related gene; AIM-g: autophagy signature-based *IDH1* mutation and grade model; DCA: decision curve analysis; DEG: differentially expressed gene; ESTIMATE: estimation of stromal and immune cells in malignant tumor tissues using expression data; GO: gene ontology; GSEA: gene set enrichment analysis; ICG: immune checkpoint gene; ROC: receiver operating curve; RS: risk score; WGCNA: weighted gene coexpression network analysis.

In an attempt to optimize the current glioma classification for prognosis, this research was mainly survival-oriented (Figure 1). A total of 881 glioma patients were included in this research (Table 1).

### 3.1. Construction of WGCNA with ATGs

**Figure 2:**
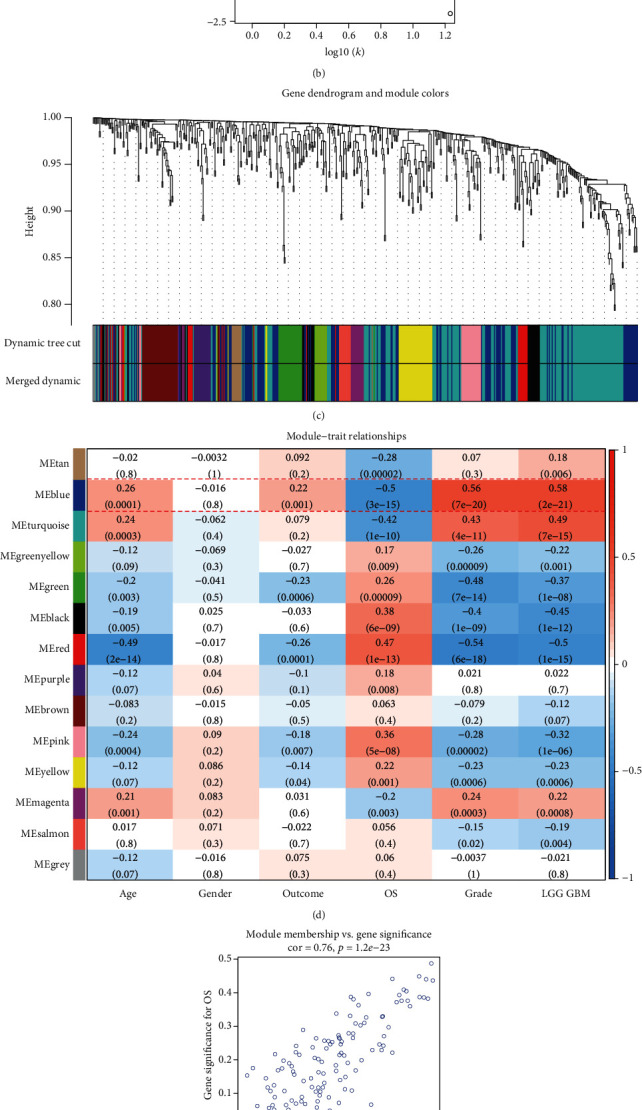
ATG signature development using WGCNA and Cox regression. (a) Soft-threshold setting for a scale-free network, the scale-free fit index was shown in the left panel and the mean connectivity was displayed in the right. (b) Scale-free plot for scale-free topology check when power was set to be 4. (c) Hierarchical clustering dendrogram of ATGs based on TOM and coexpression gene modules assigned with colors. (d) Gene module-trait relationship heat map, upper values indicated associations between module eigengenes (MEs) and clinical traits, lower values in brackets represented *p* value for the correlation analysis. (e) Scatterplot of gene significance (GS) for OS vs. module membership (MM) in the blue module, the correlation coefficient and *p* value were listed above. (f) Forest plot for the ATG signature established by Cox regression.

There were 598 ATGs assembled for this study from the MSigDB and the HADb (Table S1). It remained 548 genes for WGCNA after mapping with genes in the processed 221 glioma samples from the GEO cohort. The soft threshold power (*β*) was set as four to build an approximate scale-free topology (*R*^2^ = 0.87) (Figures 2(a) and 2(b)). As topological overlap matrix (TOM) was calculated, similarly expressed ATGs were hierarchically classified to build 14 modules using a dynamic tree-cutting algorithm (Figure 2(c)). No merged modules were observed under the merging threshold of 0.20. The 14 modules were thus identified for clinical traits correlation analysis.

### 3.2. Development of the Autophagy Signature

Module eigengenes (MEs) representing their modules were bridged to clinical data. It revealed from the module-trait relationship heat map that the blue module, consisted of 119 genes, was of interest regarding significantly close relations with patients' OS (*r* = −0.5, *p* = 3*e* − 15) (Figure 2(d)). The correlation between module membership (MM) and gene significance (GS) was also checked in the blue module (cor = 0.76, *p* = 1.2*e* − 23) (Figure 2(e)). The whole gene set of the blue module was thus selected and screened by univariate Cox regression analysis.

Only three genes, *MUL1*, *NPC1*, and *TRIM13*, of all 67 univariate screened were able to build the multivariate Cox regression model under the proportional hazards (PH) assumption (*MUL1* HR, 3.9615, 95% CI = 2.4642 − 6.369, *p* < 0.001; *NPC1* HR, 1.4957, 95% CI = 1.1389 − 1.964, *p* = 0.00379; *TRIM13* HR, 0.6292, 95% CI = 0.4888 − 0.810, *p* < 0.001) (Figure 2(f), Figure S1(a), Table S2). The autophagy signature was adjusted to be an independent risk indicator (Figure S1(b)). Hereby, autophagy risk signature was acquired and the signature risk score was calculated as follows:

(1) $$\begin{array}{c} \text{Riskscore}=1.3766\times MUL1+0.4026\times NPC1-0.4633\times \text{TRIM}13. \end{array}$$

### 3.3. Validation of the Autophagy Signature

**Figure 3:**
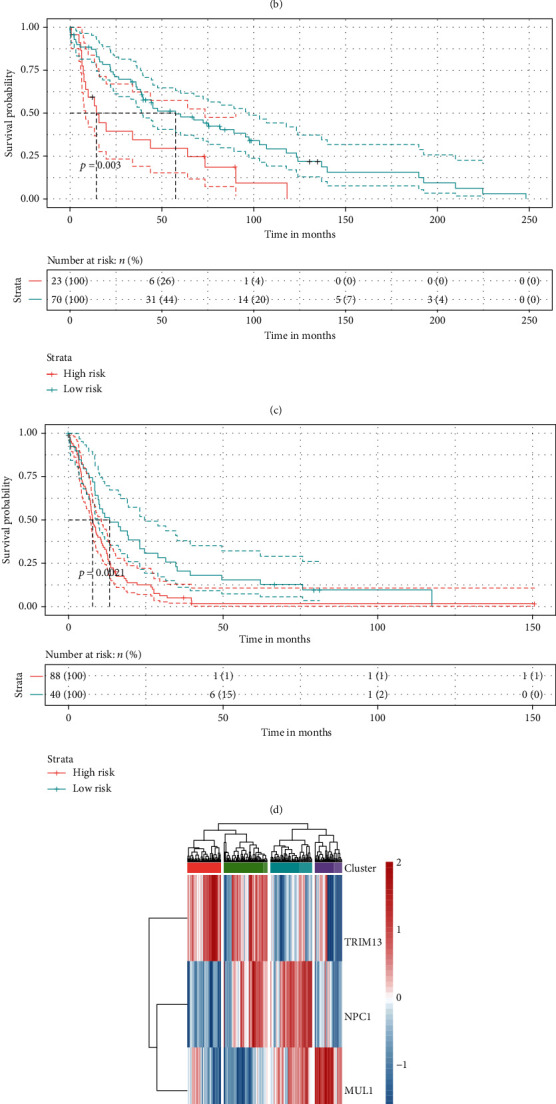
The autophagy signature member expression and survival analysis in the GEO cohort. (a) Heat map of the expression of the three-ATG signature members along with risk score. (b) Kaplan-Meier survival curve of the risk groups divided by the signature risk score median in glioma, (c) LGG, and (d) GBM. (e) Heat map of the four clusters grouped by the expression of the signature members. (f) Kaplan-Meier survival curve of the clusters.

Internal and external validation of the autophagy risk signature mainly relied on survival analysis and ROC. To start with, the expression level of the three-ATG signature was investigated (Figure 3(a)). The growing expression of *MUL1* and *NPC1* was observed with risk score increasing, whereas *TRIM13* behaved oppositely. It showed that the high-risk group had less optimistic OS than the low-risk one when the GEO cohort of glioma was subdivided with the median of the risk score (*p* < 0.0001) (Figure 3(b)). The fact that low-risk group held better survival estimation was further detailed by the survival curves in LGG (*p* = 0.003) and GBM (*p* = 0.0021) subgroups from the GEO cohort (Figures 3(c) and 3(d)).

Considering the potential grouping value of the autophagy risk signature, the GEO cohort was clustered into four groups based on the expression profile of the signature ATGs (Figure 3(e)). As depicted in the survival curves, four GEO cluster groups exhibited different OS status, which characterized by the poorest OS of the GEO clusters 3 and 4 (median OS at around 8 months) contrasted to the clusters 1 and 2 (median OS at around 34 months) (*p* < 0.0001) (Figure 3(f)). It indicated that more in-depth researches were required.

**Figure 4:**
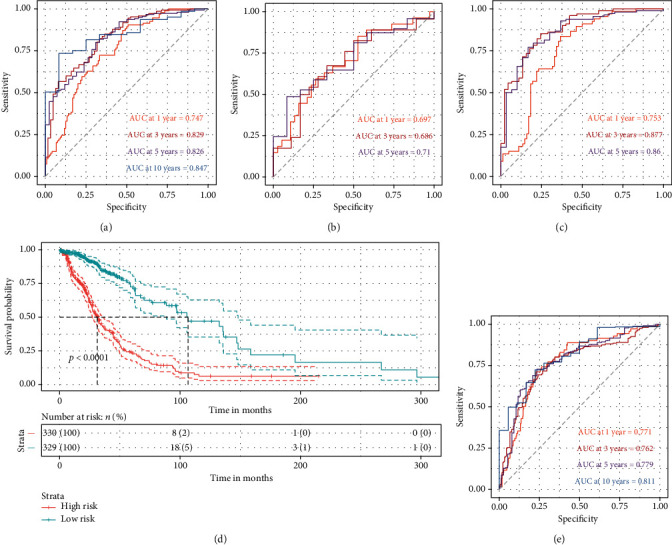
The internal and external validation of the autophagy signature with ROC and survival analysis. (a) Time-dependent ROC of the autophagy signature model trained by the whole GEO cohort at 1-, 3-, 5-, and 10-year time points. (b) Time-dependent ROC of the autophagy signature trained by the training set at 1-, 3-, and 5-year time points. (c) Time-dependent ROC of the training set-trained autophagy signature model tested by the testing set. (d) Kaplan-Meier survival curve of the risk groups divided by the signature risk score median in the TCGA cohort. (e) Time-dependent ROC of the autophagy signature model in the TCGA cohort as validation.

The discrimination of the signature was quantified by the area under the curve (AUC) of 0.747, 0.829, 0.826, and 0.847 for 1-, 3-, 5-, and 10-year OS, respectively, in the GEO cohort (Figure 4(a)). Besides, the GEO cohort was randomly cut into a training set and testing set at a ratio of 3 : 7 for the internal validation. The 1- and 3-year OS AUC of the autophagy signature in the training set were measured to be 0.697 and 0.686, while 5-year OS AUC was 0.71 (Figure 4(b)). It revealed that 1-, 3-, and 5-year OS AUC was 0.753, 0.877, and 0.86 in the testing set, respectively (Figure 4(c)).

To make it more solid, the autophagy signature was tested in the TCGA cohort of 660 gliomas as well. It was performed in the same way as in the GEO cohort with the identical risk score algorithm. The low-risk group was again proved to be favorable (*p* < 0.0001) (Figure 4(d)). Although the AUC for the 1-, 3-, 5-, and 10-year OS prediction were 0.771, 0.762, 0.779, and 0.811, it manifested autophagy signature efficacy as an indispensable facet for OS prediction (Figure 4(e)).

### 3.4. *IDH1* Mutation Involved in the OS of Autophagy Signature-Based Clusters

**Figure 5:**
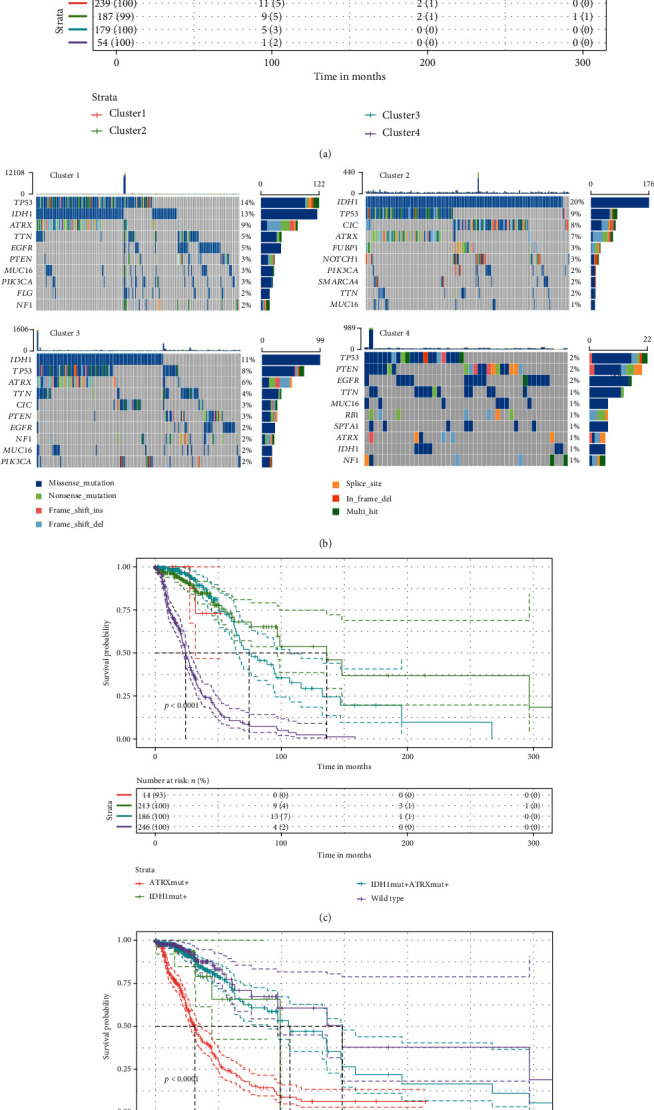
Cluster analysis and mutation profile investigation in the TCGA cohort. (a) Kaplan-Meier survival curve of the clusters in the TCGA cohort. (b) Oncoplots for mutation profiles of 901 samples acquired from the “TCGAmutations” package, categorized by the same clusters as in survival curve plot, each cluster was labeled for illustration. (c) Kaplan-Meier survival curve of the *ATRX* mutant only, *IDH1* mutant only, *ATRX* and *IDH1* double mutants, and wild type groups in the TCGA cohort. (d) Kaplan-Meier survival curve of the ATG signature risk groups with or without *IDH1* mutant in the TCGA cohort.

Intrigued by the autophagy signature-based clusters' disparate OS previously, the clusters depending on the signature expression level also showed distinct survival in the TCGA cohort (*p* < 0.0001) (Figure 5(a), Figure S1(c)). In this case, the cluster 4 displayed the worst OS of all. Another point worth noticing was the survival cures of the clusters 1 and 3 intertwined with each other. Somatic mutation profile was used to seek possible answers concerning its prevalence in glioma classification. It was revealed by the oncoplots that the cluster 4 with the poorest OS harbored drastically incompatible somatic mutation patterns in the clusters, which represented by the mutations of *TP53* 2%, *PTEN* 2%, and *EGFR* 2% (Figure 5(b)). However, the clusters 1 and 3 were characterized by the mutations of *IDH1*, *TP53*, and *TTN*. *IDH1* and *ATRX* mutants were chosen to be studied for their inclusion in the current WHO glioma classification.

It turned out that the wild type was not beneficial for OS in the TCGA cohort, whereas the *IDH1* mutant only or the *IDH1* and *ATRX* double mutants held better chances to survive (*p* < 0.0001) (Figure 5(c)). One could even conclude that the *IDH1* mutant only might benefit patients most under the present conditions. As confirmation, the *IDH1* mutant only “rescued” the both high- and low-risk group (*p* < 0.0001) (Figure 5(d)). It was concluded that the *IDH1* mutant played a pivotal role in the OS of autophagy signature-based clusters.

### 3.5. *IDH1* Mutation Links to the Signature Risk Score

**Figure 6:**
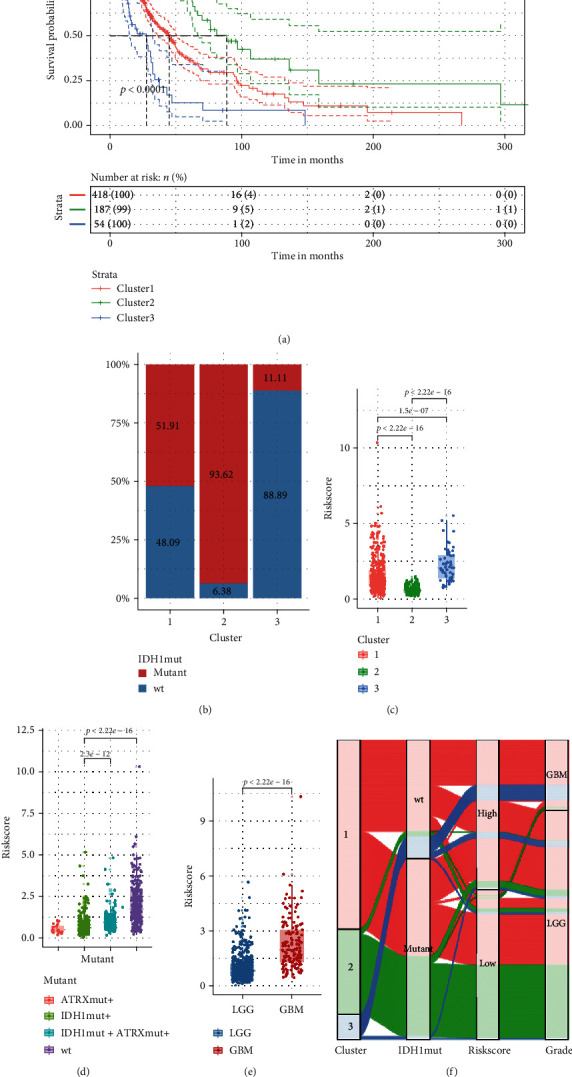
The merged clusters in the TCGA cohort and the links to risk score. (a) Kaplan-Meier survival curve of the merged clusters based on similar survival and mutation status in the TCGA cohort. (b) Bar plot for illustration of the varying IDH1mutant/wild type ratio in the merged TCGA clusters. (c) Boxplot of the merged TCGA cluster with different risk scores. (d) Box plot of the detailed *ATRX*/*IDH1* mutation groups' relation with risk score. (e) Boxplot of pathological grade linking to risk score. (f) Sankey diagram as clarification for the autophagy signature grouping value improvement by incorporating *IDH1* mutation in the final model.

The clusters 1 and 3 in the TCGA cohort were merged because of the similar *IDH1* mutation ratio and the entangled survival curves. A success was manifested by the Kaplan-Meier curves of the new three clusters stretching separately (*p* < 0.0001) (Figure 6(a)). It was inspiring that the higher the *IDH1* mutant ratio the longer OS one cluster would have in the TCGA cohort (Figure 6(b)). The barely survived cluster 3 was also observed to harbor the least amount of *IDH1* mutations (cluster 3 median OS at 27.6 months, *IDH1* mutant 11.11%; cluster 1 median OS at 44.9 months, *IDH1* mutant 51.91%; cluster 2 median OS at 88.7 months, *IDH1* mutant 93.62%). The hypothesis that the *IDH1* mutant might be positively tied to the signature-based clusters' OS was further evidenced by the GEO cohort (Figure S1(d) and (e)).

Additionally, the cluster 3 carried the significantly highest risk score with the clusters 1 and 2 following (clusters 1 and 2, *p* < 2.2*e* − 16; clusters 1 and 3 *p* = 1.5*e* − 07; clusters 2 and 3, *p* < 2.2*e* − 16) (Figure 6(c)). And the *IDH1* mutant only was slightly lower than both *IDH1* and *ATRX* mutation regarding the risk score (*p* = 2.3*e* − 12) (Figure 6(d)). As for the pathological grade, LGG held a lower risk score than GBM (*p* < 2.22*e* − 16) (Figure 6(e)). *IDH1* mutant was thus identified to serve as an affiliated classifier for better performance of the autophagy signature (Figure 6(f)).

### 3.6. Functional Enrichment Analysis of the Autophagy Signature

**Figure 7:**
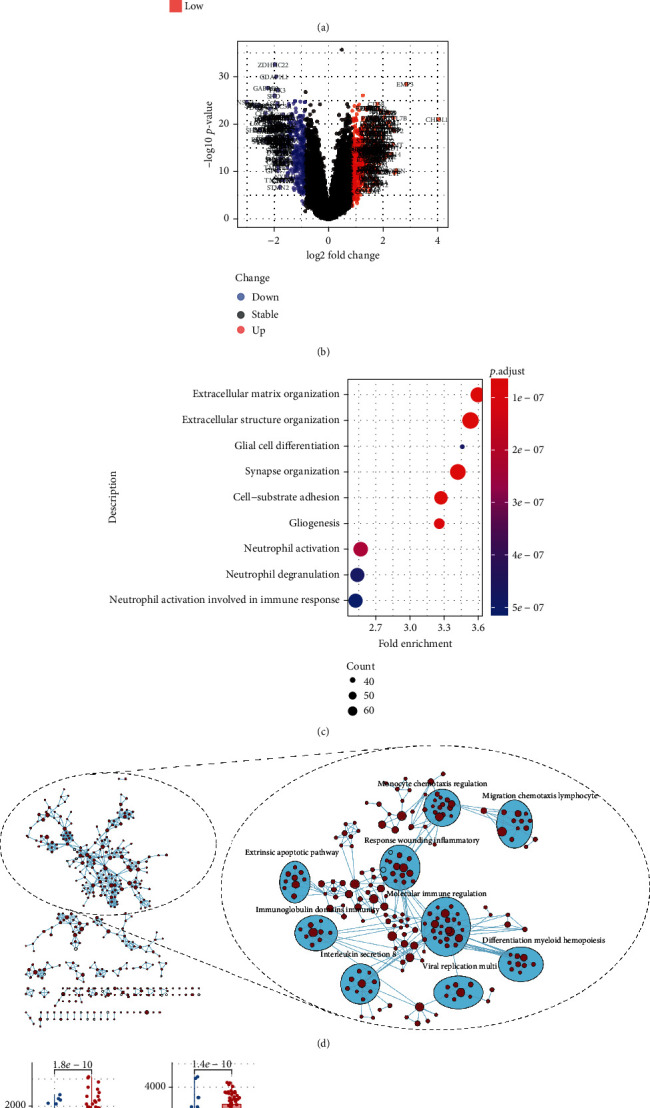
The functional enrichment analysis and immune statue estimation. (a) Heat map of the entire GEO microarray genes bifurcated by the signature risk score median into high- and low-risk groups. (b) Volcano plot of the differentially expressed genes between the high- and low-risk groups, the cutoff value was calculated as 0.85. (c) Bubble plot for the top 9 results of the GO analysis of the DEGs between the two risk groups. (d) Enrichment map auto-annotated for the representation of the GSEA results with ∣NES | >1, NOM *p* value < 0.05, and FDR *q* value < 0.25. (e) Boxplot for the estimation of the immune and stromal status of the two risk groups in the GEO cohort. (f) Circos plot for representation of the correlations among risk score and immune checkpoint genes.

It helped to interpret the function of the autophagy risk signature by performing GO analysis in the risk groups from the GEO cohort. The risk score was able to well-bifurcate the GEO cohort into two risk groups (Figure 7(a)). There were 513 up-genes and 348 downregulated genes found as DEGs between the two risk groups (Figure 7(b)). DEGs were then analyzed by GO analysis (Table S3). Interestingly, three of the top nine GO biological process (BP) enrichment results displayed neutrophil-related, such as “neutrophil activation involved in immune response” with 2.51-fold enrichment, “neutrophil degranulation” with 2.52-fold enrichment, and “neutrophil activation” with 2.55-fold enrichment (Figure 7(c)).

For further confirmation, the whole gene sets of high- and low-risk groups were used to run through GSEA with the GO biological process gene set (Table S3). It shared with the GO results that a large portion of the GSEA enrichment map automatically annotated was pertinent to immunity, like “molecular immune regulation,” “monocyte chemotaxis regulation,” and “migration chemotaxis lymphocyte” (Figure 7(d)). The functional enrichment analyses were thus leading a path to immune response, which might contribute to differentiating the autophagy risk levels.

### 3.7. ESTIMATE the Immune Status of the Autophagy Signature-Based Risk Groups

The immune-focused inquiry to autophagy signature-based risk groups was conducted by the ESTIMATE analysis in the GEO cohort. Consistent with the previous findings, it exhibited higher stromal score (presence of stroma, *p* = 1.8*e* − 10), more immune score (immune infiltration, *p* = 1.4*e* − 10), and increased ESTIMATE score (integral score, *p* = 2.6*e* − 11) in the high-risk group (Figure 7(e), Figure S1(f)). And the known 47 immune checkpoint genes (ICGs) were used to match with the genes in this cohort and tested with the risk score for possible relations. It remained 16 ICGs of significance (*p* < 0.05) with relatively strong relations (*r* > 0.2) to the risk score, of which CD200 (*r* = −0.56), LAIR1 (*r* = 0.535), CD44 (*r* = 0.53), and CD276 (*r* = 0.51) were ranked at the top (Figure 7(f)).

### 3.8. Development and Validation of AIM-g Nomogram

**Figure 8:**
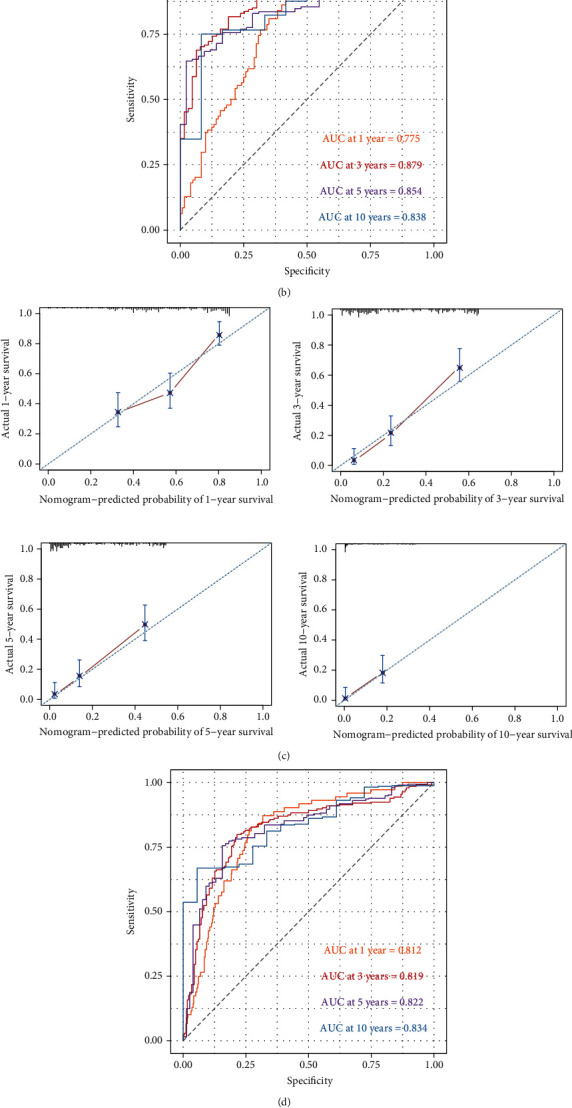
The AIM-g nomogram validations and comparisons with the WHO model. (a) Nomogram for the AIM-g model. (b) Time-dependent ROC of the AIM-g model at 1-, 3-, 5-, and 10-year time points in the GEO cohort. (c) Calibration curves of the AIM-g model for the prognostication accuracy assessments at various time points. (d) Time-dependent ROC of the AIM-g model in the TCGA cohort. (e) Time-dependent ROC of the comparisons between the AIM-g model and the WHO model at 1-, 3-, 5-, and 10-year time points. (f) DCA for the clinical utility evaluation, and comparisons between the AIM-g and the WHO model.

The autophagy risk signature, *IDH1* mutation, and tumor grade factors were adjusted and checked to be independent indices for prognosis prediction (Figure S2(a)). Since *IDH1* mutation could perfect the autophagy signature, it would improve the performance of the autophagy signature if *IDH1* mutation was included. It integrated the autophagy signature, *IDH1* mutation, and grade to build the final AIM-g nomogram in the GEO cohort (Figure 8(a)). The AIM-g model satisfied the PH assumption (Figure S2(b)). The time-ROC curves were again employed for validation, and 1-, 3-, 5-, and 10-year OS AUC was 0.775 (95% CI = 0.713 − 0.836), 0.879 (95% CI = 0.832 − 0.926), 0.854 (95% CI = 0.800 − 0.907), and 0.838 (95% CI = 0.735 − 0.941), respectively (Figure 8(b)). The 1-, 3-, 5-, and 10-year calibration curves were aligned with the standard line (Figure 8(c)). It was further verified in the TCGA cohort (1-year AUC = 0.812, 95% CI = 0.763 − 0.861; 3-year AUC = 0.819, 95% CI = 0.771 − 0.868; 5-year AUC = 0.822, 95% CI = 0.766 − 0.878; 10-year AUC = 0.834, 95% CI = 0.760 − 0.908) (Figure 8(d)).

In the end, the AIM-g nomogram was evaluated by challenging the current WHO glioma classification in the GEO cohort. It displayed a significantly higher AUC of the AIM-g at 1 year than the AUC of the WHO model being 0.74 (*p* = 0.033) (Figure 8(e)). The AIM-g AUC at 3 years was 0.879, significantly more discriminative than the WHO AUC at 3 years being 0.847 (*p* = 0.006). Moreover, the 5-year AIM-g AUC was 0.854 contrasted to 0.81 of the WHO AUC (*p* < 0.001). It also displayed the contrasting discrimination capability of the two 10-year ROC curves (10-year AIM-g AUC = 0.838; 10-year WHO AUC = 0.777, *p* < 0.001).

To clarify the net benefits for patients using this AIM-g nomogram, DCA was reasonably applied. The patients with 1-year survival probability between 0.2 and 0.5 would reap more net benefits if they selected the AIM-g model rather than the WHO model (Figure 8(f)). It showed that the AIM-g nomogram would be more advisable if a patient's 3-year survival probability was in the range of 0.2 to 0.65. The AIM-g model would also be favorably chosen when one's 5-year survival probability was within the range of 0.1 to 0.55. For the 10-year prognostication, the AIM-g model was still more beneficial.

## 4. Discussion

Autophagy was a highly conserved catabolic process throughout mammalian cells for rapid adaption to hush environment alterations. Extensive literature reported that inhibition of autophagy might sensitize glioma patients to chemotherapy [30]. However, it remained controversial that whether to inhibit protective autophagy or indulge overactive autophagy towards autophagic cell death would improve clinical outcomes [31, 32]. Even though the impairment of autophagy probably boosted the cytotoxicity of antitumor drugs, it would perturb antitumor immune responses supported by the evidence [33]. The systemic analysis of the ATGs for glioma prognostic value might not only invigorate the current glioma subtypes for prognosis from autophagic perspective but also motivate investigations on autophagy-related targets and biomarkers.

The autophagy signature constituted by *MUL1*, *NPC1*, and *TRIM13* was built at the start of this study. It was encouraging that the *IDH1* mutant involved in the autophagy signature-based survival prediction making another element for an integrative prognosis model. Considering the multifaceted possibility of the current glioma classification for improved practical application, the AIM-g nomogram was managed to attain the idea. A medical nomogram was a visual tool for individualized prognostic model [34]. After validations and evaluations, the AIM-g nomogram could be viewed as a promising and applicable model fitting such separate and “bipolar” glioma prognosis.

It was reasonable to apply WGCNA to the establishment of the ATG risk signature. WGCNA was capable of attending to coexpression genes and connecting the gene modules to clinical traits in the meantime so that the study could handle candidate genes more comprehensively than genes simply screened out by regression models. Though less perfectly associated with the OS (*r* = −0.5), it executed dimension reduction successfully and smoothed the process of the signature building with biological and statistical significance (Figures 2(d) and 2(e)). And the established three-gene signature justified the point of using WGCNA. It turned out to be robust that the ATG signature showed valid discrimination in both the internal and the external validations (Figures 4(a), 4(c), and 4(e)).

The robustness of the autophagy signature should probably be attributed to its members. *MUL1* and *NPC1* were represented as hazard factors whereas *TRIM13* exhibited protective (Figure 2(f)). MUL1 (Mitochondrial E3 Ubiquitin protein Ligase) was also known as Mitochondrial-Anchored Protein Ligase (MAPL) acting in the mitochondrial fission regulation via mitofusins, the formation of mitochondrial-derived vesicles to peroxisomes, and the proapoptosis via Drp1 SUMOylation at enhanced ER-mitochondrial contact [35]. Consistently, MAPL was identified to suppress NLRP3 inflammasome activation as SUMO-E3 ligase [36]. NPC1 (Niemann-Pick C1), one of the components of cholesterol exporting system from the lysosome, would result in the accumulation of cholesterol and glycolipids, called Niemann-Pick disease type C (NPC), if mutated [37]. It was found that NPC1 inhibited mTORC1 activation and growth signaling by binding to lysosomal protein upon cholesterol depletion [38]. TRIM13 (Tripartite Motif containing 13) stabilized p53 after ionizing radiation by ubiquitylating MDM2 and thus induce apoptosis due to its ubiquitylation ligase property [39]. Its ubiquitylation unsurprisingly initiated the autophagic flux of the p62-TRIM13 [40]. Taken together, it would still take more effort to comprehensively understand the role of each signature member in the context of glioma, which was restrained by the limited studies currently.

Based on the varying expression of the signature genes in each patient, the cohort was grouped into clusters to explore the stratification value of the signature (Figure 3(e), Figure S1(c)). The cluster 3 identified by the high expression of *MUL1* and *NPC1* and the low expression of *TRIM13* had the poorest OS among the GEO clusters (Figures 3(e) and 3(f)). It could also be deluded that the prognosis of high expression of *MUL1*and *TIRM13* was similar to the prognosis of high *NPC1* from the results (Figure 5(a), Figure S1(c)). It would be more optimistic if one negatively expressed *MUL1* and *NPC1* but showed a high expression of *TRIM13*. However, the entangled survival curves depicted in the Figures suggested additional exploration.

*IDH1* mutation taking part in the enhanced OS of the signature clusters was unexpectedly exposed (Figures 6(a) and 6(b), Figures S1(d) and (e)). Though it was assertive to conclude cause-effect relationships in a retrospective study, the previous study supported that *IDH1* mutant induced autophagy [41]. Specifically, it was the *IDH1* mutant stable U87MG cells but not the mutant glioma tissue derived from patients that exhibited increased light chain 3 phosphatidylethanolamine conjugate (LC3-II) conversion. Additional evidence from Tateishi et al. suggested that *IDH1* mutant reduced nicotinamide adenine dinucleotide (NAD+) level, of which depletion would initiate autophagy and ensuing cell vulnerability [42].

Immune responses might also be part of discerning the autophagy signature-based risk groups except for the risk score (Figures 7(c) and 7(d)). More immune cells were estimated to infiltrate in the tissue of high-risk patients, which was shared with a GBM research (Figure 7(e), Figure S1(f)) [43]. It could be explained as either the immune compensation for the “hazard” autophagy or the overexpression of ATGs because of more active immune responses. It would also replenish the statement that the interplay of autophagy and immunity inevitably impacted on treatment responses [32, 44]. CD276 (B7-H3) was demonstrated, as a member of the B7 family, to be positively correlated with the ATG risk score (Figure 7(f)). An article concluded similarly that B7-H3 regulating basal autophagy resulted in poor responses to radiation in gastric cancer [45]. In the light of immune checkpoint inhibitors prevailing in glioma, B7-H3 inhibitors might emerge as a synergistic therapeutic approach conjugated with personalized manipulation of autophagy [46].

The final AIM-g nomogram would be preferentially considered in the future for its well-defined prognostic value. It presented reliable discrimination with decent accuracy and broader practical utility (Figures 8(b)–8(d) and 8(f)). To emphasize feasibility and comparability, the current WHO glioma classification was modelized and simplified as two main elements, tumor grade and *IDH1* mutation, which were also the exact first two steps of the actual workflow. The superiority of the AIM-g, at least within the first two steps, might fulfill the desire to refine the glioma classification for more instructive prognosis estimation and potential targets of individual autophagy and immune therapy (Figures 8(e) and 8(f)).

Nonetheless, the AIM-g nomogram was yet to be tested and applied universally. It might be too concise to include overwhelmed indies for clarification and clinical application. The earlier literature claimed a glioma prognostic signature of two ATGs built on differentially expressed ATGs [47]. It might help uncover autophagy target genes but failed to assess discrimination, calibration, and clinical utility as a signature per se. The recent GBM-focused studies could be enlightened. However, they might either underestimate autophagy conserved entity in cells or overlook the ATG coexpression network and PH assumption when developing signatures [43, 48, 49].

## 5. Conclusions

This AIM-g model, based on presently available information, was the first autophagy signature-based nomogram model covering the entire gliomas. It systemically analyzed coexpressed ATGs with prognostic value and developed the autophagy signature. It also integrated the signature cluster-related *IDH1* mutation. High autophagy risk could mean more immune-active in the glioma as well. Lastly, an attempt to improve the current WHO glioma categorization for survival prediction was managed with theoretical triumph. To conclude, the AIM-g nomogram refined glioma classification for a more individualized and clinically applicable survival estimation and inspired potential autophagy-related therapies.

## Figures and Tables

**Table 1:** The clinical baseline table of the GEO and TCGA cohort.

	GEO cohort			TCGA cohort		
	*n* = 221			*n* = 660		
Age (years)	Median (50.34)			Median (46.64)		
	Age > 65	43	19.5%	Age > 65	95	14.4%
	Age ≤ 65	178	80.5%	Age ≤ 65	565	85.6%
Gender	Male	153	69.2%	Male	380	57.6%
	Female	68	30.8%	Female	280	42.4%
Grade	LGG	93	42.1%	LGG	505	76.5%
	GBM	128	57.9%	GBM	155	23.5%
*IDH1* mutation	Wild type	140	63.4%	Wild type	261	39.6%
	Mutant	81	36.7%	Mutant	399	60.5%
Chemotherapy	Yes	84	38.0%	Yes	459	69.6%
	No	137	62.0%	No	201	30.5%
Radiotherapy	Yes	154	69.7%	Yes	478	72.4%
	No	67	30.3%	No	182	27.6%
Overall survival (months)	OS < 12	100	45.3%	OS < 12	181	27.4%
	12 ≤ OS < 36	57	25.8%	12 ≤ OS < 36	297	45.0%
	36 ≤ OS < 60	22	10.0%	36 ≤ OS < 60	105	15.9%
	60 ≤ OS < 120	30	13.6%	60 ≤ OS < 120	59	8.9%
	OS ≥ 120	12	5.4%	OS ≥ 120	18	2.7%

## Data Availability

The data included in this study were obtained from the Gene Expression Omnibus (https://www.ncbi.nlm.nih.gov/geo) and The Cancer Genome Atlas database (https://cancergenome.nih.gov).

## Abbreviations

AUC: Area under the curve
ATG: Autophagy-related gene
DCA: Decision curve analysis
DEGs: Differentially expressed genes
ESTIMATE: Estimation of stromal and immune cells in malignant tumor tissues using expression data
GO: Gene ontology
GSEA: Gene set enrichment analysis
GS: Gene significance
GEO: Gene Expression Omnibus
GBM: Glioblastoma multiforme
*H3K27M*: H3.3 or H3.1lys27Met mutations
HADb: Human Autophagy Database
IDH: Isocitrate dehydrogenase
LC3-II: Light chain 3 phosphatidylethanolamine conjugate
LGG: Lower-grade glioma
MAPL: Mitochondrial-Anchored Protein Ligase
ME: Module eigengene
MM: Module membership
MSigDB: Molecular Signatures Database
MUL1: Mitochondrial E3 Ubiquitin protein Ligase
NAD+: Nicotinamide adenine dinucleotide
NPC: Niemann-Pick disease type C
Niemann-Pick C1: NPC1
MGMT: O6-methylguanine-DNA methyltransferase
OS: Overall survival
PH: Proportional hazards
ROC: Receiver operating curve
WGCNA: Weighted gene coexpression network analysis
TCGA: The Cancer Genome Atlas
TOM: Topological overlap matrix
TRIM13: Tripartite Motif containing 13.

## Supplementary Materials

Supplementary Materials . Table S1: a list of autophagy-related genes assembled from the databases. Table S2: candidate risk genes screened out by univariate Cox regression and PH assumption. Table S3: the GO result and GSEA result of the high- versus low-risk group. Figure S1: (a) Schoenfeld test plot for the visualization of the proportional hazard test for the ATG signature members, TRIM13, NPC1, and MUL1. (b) Forest plot for the adjustment of the ATG signature as an independent prognostic factor with the Age, Grade, and IDH1 mutation variables. (c) Heat map of the four clusters grouped by the expression of the signature members in the TCGA cohort. (d) Kaplan-Meier survival curve of the merged clusters based on similar survival and mutation status in the GEO cohort. (e) Bar plot for illustration of the varying IDH1 mutant/wild type ratio in the merged GEO clusters. (f) Boxplot for the estimation of the immune and stromal status of the two risk groups in the TCGA cohort. Figure S2: (a) Forest plot for the adjustment of the AIM-g model. (b) Schoenfeld test plot for the visualization of the proportional hazard test for the AIM-g model.
