# Supplementary material for: Development of Autophagy Signature-Based Prognostic Nomogram for Refined Glioma Survival Prognostication
Source: Biomed Res Int. 2020 Sep 4;2020:1872962. doi: 10.1155/2020/1872962 (PMC7492900; doi:10.1155/2020/1872962)
Supplement: Supplementary Materials — . Table S1: a list of autophagy-related genes assembled from the databases. Table S2: candidate risk genes screened out by univariate Cox regression and PH assumption. Table S3: the GO result and GSEA result of the high- versus low-risk group. Figure S1: (a) Schoenfeld test plot for the visualization of the proportional hazard test for the ATG signature members, TRIM13, NPC1, and MUL1. (b) Forest plot for the adjustment of the ATG signature as an independent prognostic factor with the Age, Grade, and IDH1 mutation variables. (c) Heat map of the four clusters grouped by the expression of the signature members in the TCGA cohort. (d) Kaplan-Meier survival curve of the merged clusters based on similar survival and mutation status in the GEO cohort. (e) Bar plot for illustration of the varying IDH1 mutant/wild type ratio in the merged GEO clusters. (f) Boxplot for the estimation of the immune and stromal status of the two risk groups in the TCGA cohort. Figure S2: (a) Forest plot for the adjustment of the AIM-g model. (b) Schoenfeld test plot for the visualization of the proportional hazard test for the AIM-g model. [file 1872962.f1.zip › 1872962.f1.docx]

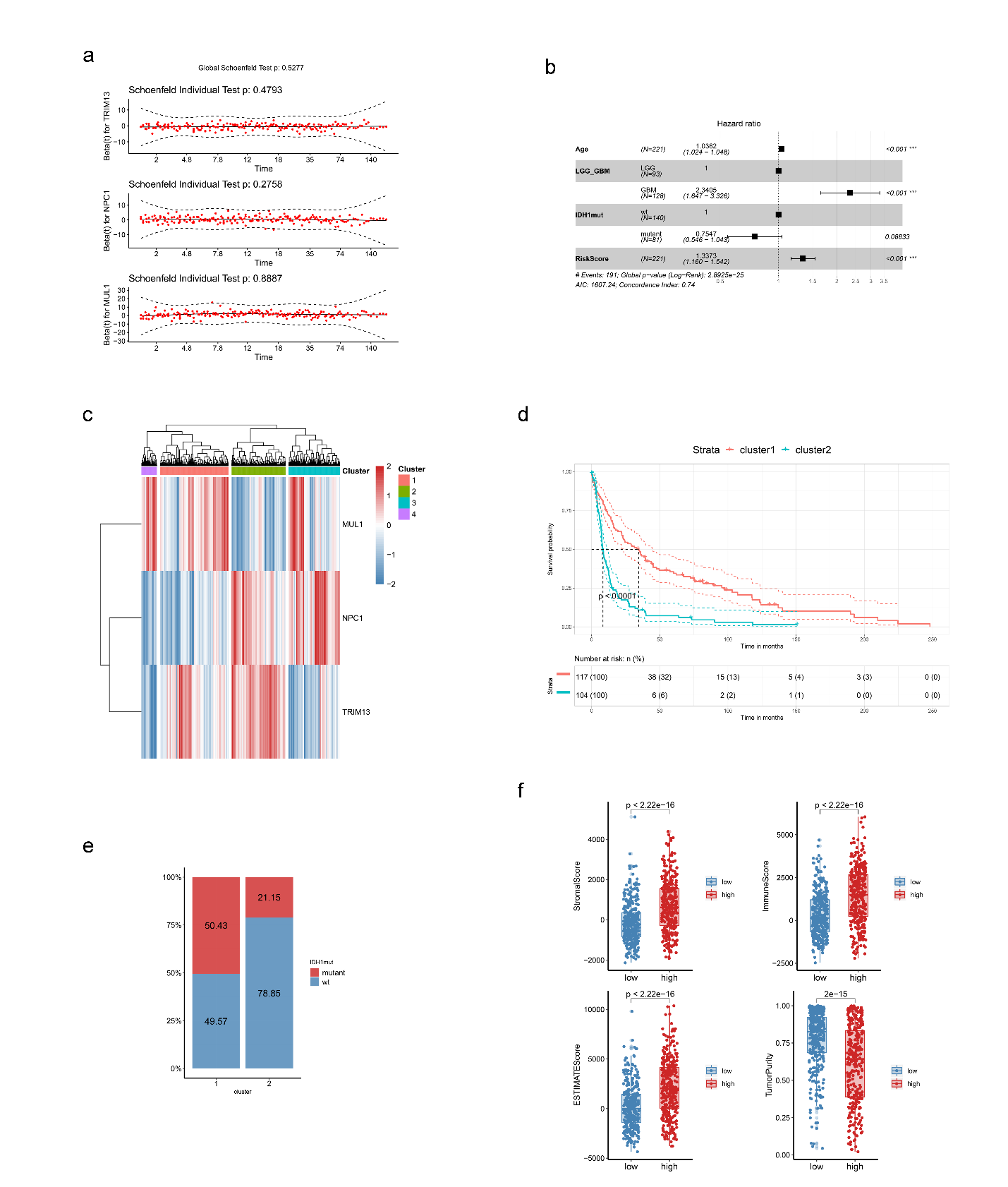


Figure S1: (a) Schoenfeld test plot for the visualization of the proportional hazard test for the ATG signature members, *TRIM13*, *NPC1*, and *MUL1*. (b) Forest plot for the adjustment of the ATG signature as an independent prognostic factor with the Age, Grade, and *IDH1* mutation variables. (c) Heatmap of the four clusters grouped by the expression of the signature members in the TCGA cohort. (d) Kaplan-Meier survival curve of the merged clusters based on similar survival and mutation status in the GEO cohort. (e) Bar plot for illustration of the varying IDH1mutant/wild type ratio in the merged GEO clusters. (f) Boxplot for the estimation of the immune and stromal status of the two risk groups in the TCGA cohort.


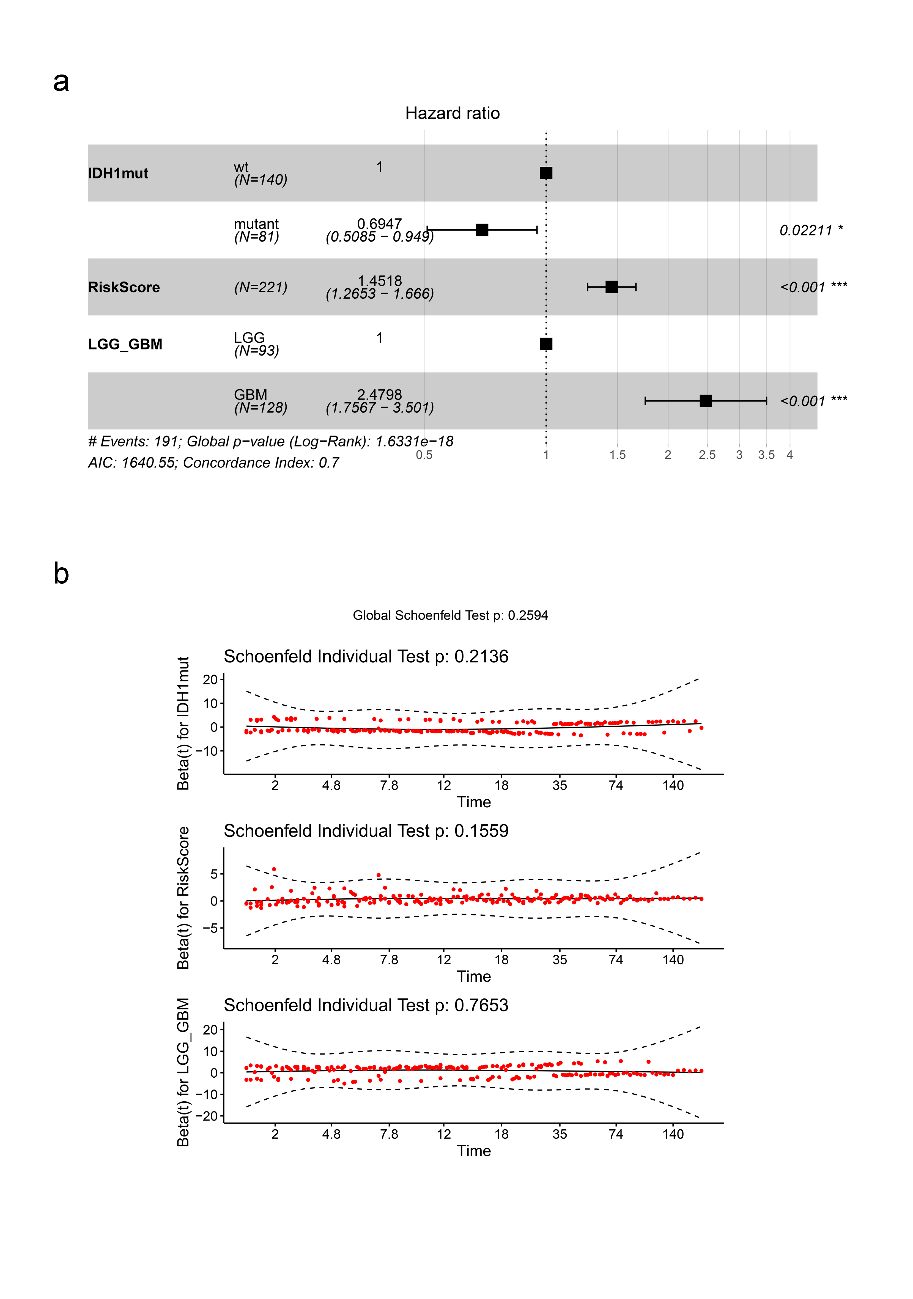


Figure S2: (a) Forest plot for the adjustment of the AIM-g model. (b) Schoenfeld test plot for the visualization of the proportional hazard test for the AIM-g model.
